# Transfer of mesenchymal stem cell mitochondria to CD4^+^ T cells contributes to repress Th1 differentiation by downregulating T-bet expression

**DOI:** 10.1186/s13287-022-03219-x

**Published:** 2023-01-24

**Authors:** Waseem Akhter, Jean Nakhle, Loïc Vaillant, Geneviève Garcin, Cécile Le Saout, Matthieu Simon, Carole Crozet, Farida Djouad, Christian Jorgensen, Marie-Luce Vignais, Javier Hernandez

**Affiliations:** 1 Institute for Regenerative Medicine and Biotherapy (IRMB), INSERM U1183, Université de Montpellier, 34295 Montpellier, France; 2 IGF, CNRS, INSERM, Université de Montpellier, Montpellier, France; 3 IGMM, CNRS, Université de Montpellier, Montpellier, France; 4 INM, INSERM, Université de Montpellier, Montpellier, France; 5 CHU Montpellier, Montpellier, France

**Keywords:** Mesenchymal stem/stromal cells, CD4^+^ T cells, Mitochondrial transfer, Immunotherapy, Autoimmunity

## Abstract

**Background:**

Mesenchymal stem/stromal cells (MSCs) are multipotent cells with strong tissue repair and immunomodulatory properties. Due to their ability to repress pathogenic immune responses, and in particular T cell responses, they show therapeutic potential for the treatment of autoimmune diseases, organ rejection and graft versus host disease. MSCs have the remarkable ability to export their own mitochondria to neighboring cells in response to injury and inflammation. However, whether mitochondrial transfer occurs and has any role in the repression of CD4^+^ Th1 responses is unknown.

**Methods and results:**

In this report we have utilized CD4^+^ T cells from HNT TCR transgenic mice that develop Th1-like responses upon antigenic stimulation in vitro and in vivo. Allogeneic bone marrow-derived MSCs reduced the diabetogenic potential of HNT CD4^+^ T cells in vivo in a transgenic mouse model of disease. In co-culture experiments, we have shown that MSCs were able to reduce HNT CD4^+^ T cell expansion, expression of key effector markers and production of the effector cytokine IFNγ after activation. This was associated with the ability of CD4^+^ T cells to acquire mitochondria from MSCs as evidenced by FACS and confocal microscopy. Remarkably, transfer of isolated MSC mitochondria to CD4^+^ T cells resulted in decreased T cell proliferation and IFNγ production. These effects were additive with those of prostaglandin E2 secreted by MSCs. Finally, we demonstrated that both co-culture with MSCs and transfer of isolated MSC mitochondria prevent the upregulation of T-bet, the master Th1 transcription factor, on activated CD4^+^ T cells.

**Conclusion:**

The present study demonstrates that transfer of MSC mitochondria to activated CD4^+^ T cells results in the suppression of Th1 responses in part by downregulating T-bet expression. Furthermore, our studies suggest that MSC mitochondrial transfer might represent a general mechanism of MSC-dependent immunosuppression.

**Supplementary Information:**

The online version contains supplementary material available at 10.1186/s13287-022-03219-x.

## Background

Conditions in which unwanted immune responses exist, such as autoimmune disorders, graft versus host disease (GVHD) and graft rejection, pose serious health problems. One of the main effectors contributing to these immune-mediated disorders are T cells. Both CD4^+^ and CD8^+^ T cells are involved. CD8^+^ cytotoxic T lymphocytes (CTL) can directly kill target cells and secrete inflammatory cytokines such as IFNγ and TNFα. CTL play an important role in the pathogenesis of GVHD, graft rejection and autoimmune diseases such as type 1 diabetes and aplastic anemia [1]. CD4^+^ Th1 effector cells mainly secrete IFNγ, activate M1 macrophages and provide help for CD8^+^ T cell responses. They are thought to be the main effectors in psoriasis and inflammatory bowel disease and play a major role in graft rejection. CD4^+^ Th17 cells produce high amounts of IL-17 and IL-22, activate epithelial cells and activate and recruit other immune cells such as neutrophils. Th17 cells are arguably the main T cell subset involved in multiple sclerosis and rheumatoid arthritis [2]. Therefore, a major challenge is to develop therapeutic strategies able to suppress these unwanted T cell responses without excessively compromising protective T cell responses against pathogens. Classical immunosuppressive drugs used to restrain these responses have profound side effects, including general immunosuppression. As an alternative, a number of strategies aiming to restore or promote immune tolerance have been recently proposed [3]. Adoptive cell therapies including antigen-specific CD4^+^ Foxp3^+^ regulatory T cells (Tregs), Chimeric Antigen Receptor Tregs, tolerogenic dendritic cells (DCs) and immunosuppressive Mesenchymal stem/stromal cells (MSCs), represent promising candidates that are currently under study [4]. Tolerogenic DCs can be loaded with relevant self-antigens and restore antigen-specific tolerance in autoimmune diseases [4]. Low numbers of functional Tregs are frequently present in autoimmune diseases patients. Infusion of expanded antigen-specific Tregs or more recently CAR-Tregs have shown their potential to stop anti-self T cell responses with no induction of general immunosuppression in pre-clinical models [4]. Finally, MSCs are able to migrate to the site of injury and inflammation exerting an immunosuppressive effect locally and are currently tested in a number of clinical trials [4].

MSCs are present in numerous adult tissues including the bone marrow, adipose tissue, placenta, umbilical cord and connective tissues. They have multipotent differentiation potential and, apart from their tissue repair capacity, also have strong immunomodulatory properties [5–8]. These immunomodulatory properties highly depend on the surrounding environment. In the steady state, MSCs may have an homeostatic role supporting survival of components of the immune system [9]. On the other hand, under inflammatory conditions, or after treatment with IFNγ or TNFα in vitro, MSCs become strongly immunosuppressive [10, 11]. Thus, due to their suppressive effects, infusion of MSCs is currently tested in a number of clinical trials to treat autoimmune diseases, including type 1 diabetes, rheumatoid arthritis, systemic lupus erythematosus, inflammatory bowels disease as well as organ rejection and graft versus host disease [5–8]. Many different immune cell subsets are potential targets of MSCs. MSCs can downmodulate the inflammatory properties of innate immune cells such as DC, macrophages, NK cells and neutrophils, and suppress pathogenic adaptive B and T cell responses in vitro and in vivo [6, 12].

MSCs can suppress the expansion of both CD4^+^ and CD8^+^ T cells [13–15]. MSCs have also been shown to inhibit the differentiation of naïve T cells into effectors and the function of effector CD4^+^ Th1, CD4^+^ Th17 and CD8^+^ T cells [16–22]. On the other hand, they induce Tregs and promote Th2 responses [16, 16, 17, 23–26]. The mechanisms via which MSCs exert their immunosuppressive effect on T cells are multiple and may vary depending on the system under study. Under inflammatory conditions, MSCs upregulate PD-L1, which dampens pathogenic CD4^+^ T cell responses upon engagement with PD-1 expressed on effector T cells [27–31]. They also express FasL that can mediate direct killing of activated T cells [32, 33]. MSCs secrete anti-inflammatory molecules and cytokines, e.g., TGF-β, IL-10, IL-1RA, hepatocyte growth factor, prostaglandin E 2 (PGE2), which can block T cell proliferation, prevent Th1 and Th17 differentiation and induce Tregs [16, 25, 34]. Pro-inflammatory cytokine-activated human MSCs express high levels of indoleamine 2,3-dioxygenase (IDO) that degrades tryptophane into kynurenine. Tryptophan deprivation and kynurenine derivatives are toxic and suppress T cell proliferation [17, 35]. Whereas murine MSCs do not express IDO, they do express inducible nitric oxide synthase (iNOS) and release nitic oxide that in turn can suppress T cell differentiation [36]. Notably, it has been recently shown that extracellular vesicles secreted by MSCs can recapitulate many of their immunosuppressive effects on T cell responses [37].

It is now well established that MSCs are able to transfer their mitochondria to many different cellular types. MSCs express Toll-like receptors and inflammation sensing receptors that allow them to sense damage and respond to tissue injury [38]. In response to elevated levels of reactive oxygen species (ROS) and mitochondrial products, MSCs have been shown to transfer mitochondria to neighboring cells in vivo and in vitro [39, 40]. This intercellular organelle exchange is most frequently mediated through tunneling nanotubes. Additionally, MSC mitochondria can be shuttled from one cell to another in extracellular vesicles released by MSCs [39–41]. The result of MSC mitochondrial transfer to injured cells is protection against damage by modulating and restoring their metabolism [39, 40, 42]. In cancer cells, mitochondria uptake from MSCs results in enhanced proliferation and invasiveness as well as resistance to cytotoxic drugs via a metabolic shift enhancing oxidative phosphorylation [39, 41].

Cells of the immune system have also been shown to be recipients of MSC mitochondrial transfer. Transfer of MSC mitochondria increased the phagocytic potential of macrophages confronted to pathogenic bacteria in vitro and in vivo [43]. Additionally, transfer of MSC mitochondria through extracellular vesicles can promote anti-inflammatory M2 macrophages with enhanced phagocytic function in a model of lung injury [44]. We have recently shown that human MSCs transfer mitochondria to human Th17 cells [45]. MSC mitochondria diminished Th17 cell proliferation as well as their effector functions and induced their conversion into Tregs [45]. MSC mitochondrial transfer promotes the stabilization of Foxp3 in human induced Tregs by enhancing the expression of the BACH2 and SENP3 stabilizers [46]. Induction of Tregs by MSC mitochondria has also been shown in a mouse model of GVHD [47]. Furthermore, mitochondrial transfer from allogenic MSCs to human Tregs improve their immunosuppressive potential [48]. Remarkably, it has been demonstrated that transfer of mitochondria isolated from MSCs to recipient cells, termed mitoception, recapitulates the suppressive effects of MSCs in co-cultures [45, 49]. Whether mitochondrial transfer from MSCs to immune cells is a general mechanism of immunosuppression and the nature of underlying mechanisms remain unknown. Here, we investigated the role of murine MSC mitochondrial transfer on the suppression of mouse CD4^+^ Th1 responses.

## Materials and methods

### Mice

C57BL/6mice were purchased from Charles River Laboratories. Balb/c HNT TCR transgenic mice express a MHC class II I-A^d^ restricted TCR specific for the influenza virus PR/8 hemagglutinin (HA) epitope 126–138 [50]. Balb/c InsHA transgenic mice express the influenza virus HA under the control of rat insulin promoter, driving its expression to pancreatic beta cells [51]. Balb/c Clone 4 TCR transgenic mice express a HA-specific MHC class I K^d^ restricted TCR [52]. Both males and females 8 to 16 weeks of age were used in all experiments. Mice were euthanized by carbon dioxide asphyxiation. Mice were bred and maintained under specific pathogen free conditions in an enriched environment at the animal facility of the Institute for Neurosciences of Montpellier Saint Eloi.

### Isolation of bone marrow derived MSCs

The procedure has been previously described [53]. Briefly, bone marrow from tibias and femurs of C57BL/6 mice were collected. Cell suspensions were seeded at a concentration of 10^6^ cells/cm^2^ in modified minimum essential Eagle's medium (MEM) supplemented with 10% heat-inactivated fetal bovine serum (FBS) (Hyclone, Thermo Fisher Scientific), 2 mM glutamine, 100 U/ml penicillin, 100 mg/ml streptomycin (Lonza, Levallois-Perret, France) and 2 ng/ml human basic fibroblast growth factor (bFGF) (R&D Systems, Lille, France). MSCs were CD29^+^, Sca1^+^, CD73^+^. Functionally, MSCs had the capacity to differentiate into adipocytes, chondrocytes and osteoblasts when cultured under specific and appropriated conditions [53].

### T cell isolation

CD4^+^ T cells were purified from the lymph nodes and spleen of HNT TCR transgenic mice using the Dynal® CD4^+^ negative isolation kit according to the manufacturer's instructions (Thermo Fisher Scientific). CD8^+^ T cells were purified from the lymph nodes and spleen of Clone 4 TCR transgenic mice using the Dynal® CD8^+^ negative isolation kit according to the manufacturer's instructions (Thermo Fisher Scientific).

### T cell activation and co-culture with MSCs

Purified CD4^+^ T cells were activated in anti-CD3 (Clone 145-2C11, BioXcell,) and anti-CD28 (Clone 37.51, BioXcell) coated 24-well plates at a density of 10^6^ cells per well in RPMI media (RPMI Media 1640 1X + Glutamax, Gibco, Life Technologies, UK) containing 10% FBS (Gibco, Life Technologies,Germany), 2 mM L-glutamine, 100 U/ml penicillin, 100 µg/ml streptomycin (Gibco, Life Technologies, USA), 50 µM beta-mercaptoethanol (Gibco, Life Technologies, USA) in the presence of 25 U/ml of mouse recombinant IL-2 (PeproTech, USA) and cultured at 37 °C, 5% CO_2_ for up to 4 days as indicated. To study proliferation, isolated T cells were labeled with 5 μM CFSE (CFSE Cell Proliferation Kit, Invitrogen, Thermo Fisher, USA) in PBS for 10 min at 37 °C and plated after washing. In some experiments, PGE2 (PeproTech, USA) was added to the T cell media at the indicated concentrations on day 0. Pooled lymph nodes from 1 to 3 mice were used to obtain T cells for each experiment and all experiments were performed in triplicate wells.

For the co-culture experiments, MSCs were either pre-treated with 10 ng/ml recombinant murine TNFα (PeproTech, USA) and 20 ng/ml of recombinant murine IFNγ (PeproTech, USA) for 48 h in MSC media (DMEM (Dulbecco's Modified Eagle Medium) 1X + Glutamax + 4.5 g/L D-Glucose + Pyruvate) or left without treatment. Cells were then harvested and 4 × 10^4^ were added per well to activated T cells in T cell media on day 0, ratio of 1:25 (MSC:T cell). In some experiments, Indomethacin (ThermoFisher, GmbH, Germany) was added to the co-cultures at a concentration of 10 µM on day 0. For trans-well experiments, 24-well plates with 0.4 μm pore size inserts were used. 5 × 10^5^ T cells were seeded in antibody coated wells and 2 × 10^4^ MSCs were seeded in the insert. To label mitochondria, TNFα and IFNγ treated MSCs were stained with the MitoTracker Red CMXRos at 500 nM (Molecular Probes, Invitrogen, USA) or MitoTracker Deep Red FM at 250 nM (Molecular Probes, Invitrogen, USA) fluorescent mitochondrial dyes according to the manufacturer’s instructions. After 24 h of culture, labeled cells were harvested, washed again extensively and added to T cells. All experiments were performed in triplicate wells.

### Isolation of MSC mitochondria and transfer to T cells (mitoception)

Transfer of MSC isolated mitochondria to T cells was performed as previously described with modifications [49]. Mitochondria were isolated from 5 × 10^5^ MSCs trypsinized in the absence of EDTA. Cells were lysed in ice-cold mannitol buffer (mannitol 210 mM, saccharose 70 mM, EDTA 1 mM, HEPES 10 mM) in the presence of a protease/phosphatase inhibitor cocktail and centrifuged at 800 g at 4 °C for 10 min to eliminate nuclei. The mitochondrial fraction pellet was recovered after centrifugation of the supernatant at 8000 g at 4 °C for 10 min. Isolated mitochondria were maintained on ice in mannitol buffer and then diluted in RPMI T cell media with 10% FBS for immediate transfer into purified CD4^+^ T cells plated in 96-well plates. Culture plates were centrifuged at 3000 g at RT for 15 min and incubated at 37 °C, 5% CO_2_ for 12 h. Then, T cells were harvested, plated in 24-well plates, activated and cultured in T cell media as described above. Control mock mitocepted T cells underwent the same procedure without the addition of mitochondria. In some experiments, PGE2 was added to mitocepted T cells at the indicated concentrations. In other experiments, 0.5 ml out of 2 ml of the T cell media used in each well was replaced by 0.5 ml of MSC conditioned media during the activation/culture period. Conditioned media was recovered from MSCs that were previously activated with TNFα and IFNγ, harvested, washed, plated and cultured for 2 days. In parallel experiments, MitoTracker-stained MSC were used as a source of mitochondria and labeled mitochondria were transferred to T cells. Mitoception efficiency was verified by flow cytometry analysis of T cells 12 h later. All experiments were performed in triplicate wells.

### Adoptive transfer experiments

Equal numbers (3 × 10^6^ cells/mouse) of day 3 activated HNT CD4^+^ T cells, or day 3 activated HNT CD4^+^ T cells in the presence of MSCs, and purified naïve Clone 4 CD8^+^ T cells were injected i.v. into InsHA mice that had been sublethally irradiated (3 Gy) 24 h before in a RS2000 irradiator (RadSource, USA). Some mice also received 10^6^ MSCs i.v. the day of T cell transfer and 5 days later. Gender and age matched individuals were randomly assigned to control and experimental groups. Adoptively transferred mice blood glucose levels were monitored using a glucometer (AccuCheck). Mice were considered diabetic when blood glucose levels were > 300 mg/dl for 2 consecutive points. Measurements were performed at the same time of the day and in the same order. Diabetic mice were monitored daily and euthanized at first signs of distress. All treated animals were included in the analysis.

### Flow cytometry analysis

For phenotyping, T cells were harvested and stained in PBS 1X (Gibco, Life Technologies Europe B.V., UK) containing 2% FBS and 0.02% sodium azide at 4 °C for 20 min with the following mAbs: anti-CD4-PerCP-Cy5-5, anti-CD25-PE-Cy7 (BD Pharmingen™, USA) and anti-CD40L-PE (eBioscience, USA). To assess transcription factor expression, staining was performed using the Fixation and Permeabilization Kit (eBioscience, USA) according to manufacturer’s instructions with anti-FoxP3-APC (eBioscience, USA) and anti-Tbet-eFlour660 (BD Pharmingen™, USA). To assess cytokine production, harvested T cells were stimulated for 4 h with 50 ng/ml phorbolmyristate acetate (PMA) (Sigma-Aldrich), 0.5 µg/ml ionomycin (Sigma-Aldrich) in the presence of 10 µg/ml brefeldin A (Sigma-Aldrich, USA). Then, intracellular cytokine staining was performed using the Cytofix/Cytoperm Kit (BD PharMingen) with anti-IL-2-APC, anti-INFγ-PE or anti-INFγ-APC (BD Pharmingen™, USA). Isotype-matched antibodies were used as controls. To assess mitochondrial transfer, T cells that had been co-cultured with MitoTracker labeled MSCs or that had received labeled mitochondria were harvested after 12 h, washed and immediately analyzed. Cells were analyzed on a FACSCanto II apparatus using Diva or FlowJo software (BDB).

### Confocal microscopy

Naïve isolated HNT CD4^+^ T cells were activated, labeled with CFSE and cultured with MitoTracker Red CMXRos or MitoTracker Deep Red FM-labeled MSCs. T cells were then harvested and seeded on glass slides, fixed with paraformaldehyde 3.7% and mounted with Prolong Gold. Images were taken with a confocal laser microscope (Zeiss LSM880 or Leica TCS SP8-X). 3D reconstruction was done using the Imaris Bitplane or the LAS X 3D visualization advanced module software.

### Statistical analysis

Values are represented as means ± SEM. Statistical tests were performed using GraphPad Prism. Comparisons were made using two-tailed Mann–Whitney *U*-test. Multiple comparisons were made using one-way ANOVA. *P* values were considered significant at *P < 0.05, **P < 0.01, ***P < 0.001, and ****P < 0.0001.

## Results

### MSCs suppress the differentiation of HNT CD4^+^ T cells into effector cells

**Fig. 1:**
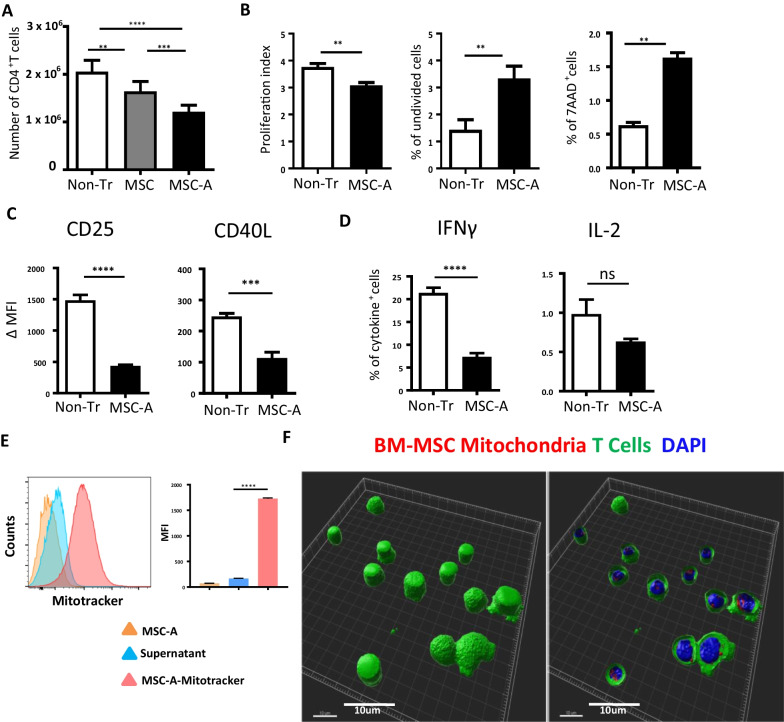
Allogeneic bone marrow-derived MSCs suppress HNT CD4^+^ T cell responses and transfer mitochondria. **A** Naïve purified HNT CD4^+^ T cells were activated with anti-CD3 and anti-CD28 mAbs and co-cultured with C57BL/6 bone marrow-derived MSCs (MSC) or TNFα/IFNγ-activated MSCs (MSC-A). Control activated HNT CD4^+^ T cells were left untreated without any MSCs (Non-Tr). After 4 days, CD4^+^ T cells were harvested and enumerated. Absolute numbers of cultured HNT CD4^+^ T cells are shown. Values are represented as mean ± SEM. Data from 5 independent experiments is presented. **B** CFSE-labeled HNT CD4^+^ T cells were activated and co-cultured with MSC-A or left untreated. At day 4, T cells were harvested and CFSE fluorescence analyzed by FACS. Viability was assessed by 7-AAD uptake. Proliferation index was calculated using FlowJo software. Data from 5 independent experiments is presented. Values are represented as mean ± SEM. **C** Day 4 activated HNT CD4^+^ T cells cultured in the presence or absence of MSC-A were harvested and expression of CD25 and CD40L activation markers was assessed by FACS. Mean fluorescence intensity (MFI) minus that of the isotype controls is indicated. Data from 4 independent experiments is presented. Values are represented as mean ± SEM. **D** Day 4 activated HNT CD4^+^ T cells were restimulated with PMA and ionomycin in the presence of brefeldin A and production of intracellular IFNγ and IL-2 was assessed by FACS. Percentage of cytokine-producing T cells is indicated. Data from 4 independent experiments is presented. Values are represented as mean ± SEM. **E** HNT CD4^+^ T cells were activated and cultured with MSC-A or Mitotracker Deep Red-labeled MSC-A. As control HNT CD4^+^ T cells were activated and cultured with the supernatant of Mitotracker Deep Red-labeled MSC-A that underwent the same process of labeling, washing and culturing time. After 12 h, harvested T cells were analyzed by FACS. Data from one representative experiment out of three is presented. Values represent MFI ± SEM. **F** HNT CD4^+^ T cells were stained with CFSE and cultured with MitoTracker Red CMXRos-labeled MSC-A for 12 h. Three-dimensional reconstruction of confocal microscopy images of T cells (left, whole cells; right, cell sections). Scale bar 10 μm

Transgenic Balb/c HNT TCR (HNT) mice express a MHC class II restricted, influenza hemagglutinin (HA) specific TCR [1, 50]. When activated in vivo, or in vitro under non-polarizing conditions, HNT CD4^+^ T cells differentiate into Th1-like IFNγ-producing cells that are capable of providing help for the differentiation of CD8^+^ T cells into effector CTL [1, 54]. We utilized allogeneic bone-marrow derived MSCs from C57BL/6 mice, already characterized [53], to assess their ability to suppress HNT CD4^+^ T cell responses in co-culture experiments. Isolated naïve HNT CD4^+^ T cells were activated with anti-CD3 and anti-CD28 mAbs in the presence or absence of MSCs at a 1:25 ratio (MSC:Tcells). After four days in culture, CD4^+^ T cells had extensively proliferated (Fig. 1A). MSCs significantly reduced CD4^+^ T cell expansion (Fig. 1A). Under the same settings, CD4^+^ T cells were incubated with MSCs that had been previously activated with TNFα and IFNγ during 48 h (MSC-A). It is well established that inflammatory cytokines promote MSC immunosuppressive effects [10]. Indeed, we found that MSC-A significantly reduced the number of anti-CD3/CD28 activated CD4^+^ T cells harvested after 4 days in culture (Fig. 1A). Therefore, we decided to use only pretreated MSCs in the next experiments. CFSE profiles of proliferating activated CD4^+^ T cells showed that they underwent up to 6 rounds of division. And although the profiles were not drastically changed, the proliferation index was significantly reduced for CD4^+^ T cells in the presence of MSC-A (Fig. 1B and Additional file 1: Fig. 1A). This reduction in the proliferation index reflects the fact that there were more undivided cells and fewer cells that underwent more than 3 rounds of division in the presence of MSC-A (Fig. 1B and Additional file 1: Fig. 1A). Additionally, we assessed the viability of proliferating cells by 7-AAD staining and found a twofold increase in the number of dying cells in the presence of MSC-A (Fig. 1B). We next investigated the CD4^+^ T cell activation status by assessing the expression of key activation markers. It is important to note that no remarkable differences were found in the size of activated cells in the presence or absence of MSCs (Additional file 1: Fig. 1A). As expected, activated CD4^+^ T cells upregulated CD25 and CD40L (Additional file 1: Fig. 1B and C). This upregulation was significantly inhibited by co-culture with MSC-A (Fig. 1C and Additional file 1: Fig. 1C). Activated CD4^+^ T cells also acquired the potential to secrete the Th1 effector cytokine IFNγ as revealed by restimulation and intracellular cytokine staining (Fig. 1D and Additional file 1: Fig. 1D and E). MSC-A greatly diminished the percentage of IFNγ producing cells (Fig. 1D and Additional file 1: Fig. 1E). On the other hand, the number of IL-2-producing HNT CD4^+^ T cells was very low and no significant differences were found in the presence or absence of MSCs (Fig. 1D and Additional file 1: Fig. 1D and E). These results demonstrate that MSC-A decrease proliferation, increase apoptosis of proliferating cells and suppress the acquisition of an effector phenotype and functionality by HNT CD4^+^ T cells upon activation mimicking an antigenic encounter.

HNT CD4^+^ T cells are able to cooperate with HA-specific Clone 4 CD8^+^ T cells in the induction of autoimmune diabetes when transferred into mice that express the HA antigen in the beta cells of the pancreas, InsHA mice, under inflammatory conditions [1, 54–57]. We have previously shown that HNT CD4^+^ T cells provide help for the differentiation of Clone 4 CD8^+^ T cells in diabetogenic CTL [1, 54, 57]. Thus, we assessed whether co-culture of HNT CD4^+^ T cells with MSC-A had an impact on their helper function in vivo. To this end, we co-transferred day 3 activated HNT CD4^+^ T cells, cultured in the presence or absence of MSC-A, along with naïve Clone 4 CD8^+^ T cells into sublethaly irradiated InsHA mice. We monitored mice for the onset of autoimmune diabetes by measuring blood glucose levels and observed no differences between the two groups (Additional file 1: Fig. 2A). However, InsHA mice that received day 3-activated HNT CD4^+^ T cells, cultured beforehand with MSC-A, along with naïve Clone 4 CD8^+^ T cells and that were additionally treated in vivo with MSCs showed a significant delay in the onset of autoimmune diabetes (Additional file 1: Fig. 2A). On the other hand, InsHA mice adoptively transferred with day 3-activated HNT CD4^+^ T cells, in the absence of MSC-A, along with naïve Clone 4 CD8^+^ T cells and treated in vivo with MSCs showed a very modest delay over control mice (Additional file 1: Fig. 2B). These results indicate that co-culture of HNT CD4^+^ T cells with MSC-A suppress their helper activity in vivo although this effect needs to be reinforced by further injection of MSCs into InsHA mice.

**Fig. 2:**
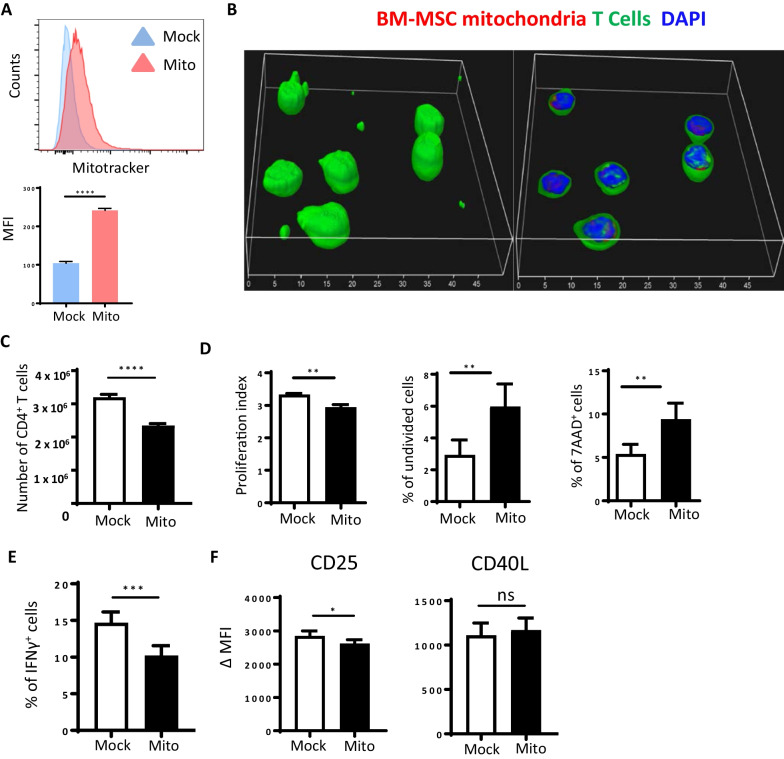
Transfer of MSC mitochondria to HNT CD4^+^ T cells inhibits their expansion and gain of effector function. **A** Isolated mitochondria from MSC-A labeled with Mitotracker Deep Red were transferred by mitoception to naïve HNT CD4^+^ T cells. Control HNT CD4^+^ T cells were mock mitocepted without mitochondria. 12 h later CD4^+^ T cells were analyzed by FACS. Data from one representative experiment out of three is presented. Values represent MFI ± SEM. **B** Isolated mitochondria from MSC-A labeled with Mitotracker Deep Red were transferred by mitoception to CFSE-labeled HNT CD4^+^ T cells. After 12 h cells were prepared for microscopy. Three-dimensional reconstruction of confocal microscopy images of T cells (left, whole cells; right, cell sections). Scale bar 10 μm. **C** Isolated mitochondria from MSC-A were transferred by mitoception to naïve HNT CD4^+^ T cells. 12 h later, mitocepted or mock mitocepted HNT CD4^+^ T cells were activated with anti-CD3 and anti-CD28 mAbs and cultured during 4 days. Absolute numbers of cultured HNT CD4^+^ T cells are shown. Values are represented as mean ± SEM. **D** Mitocepted or mock mitocepted CFSE labeled HNT CD4^+^ T cells were activated and cultured. After 4 days, CD4^+^ T cells were harvested and CFSE fluorescence analyzed by FACS. Viability was assessed by 7-AAD uptake. Proliferation index was calculated using FlowJo software. Data from 3 independent experiments is presented. Values are represented as mean ± SEM. **E** Activated HNT CD4^+^ T cells were restimulated with PMA and ionomycin in the presence of brefeldin A and production of intracellular IFNγ was assessed by FACS. Percentage of cytokine-producing T cells is indicated. **F** Expression of CD25 and CD40L in activated HNT CD4^+^ T cells. Mean fluoresce intensity (MFI) minus that of the isotype controls is indicated. Values are represented as mean ± SEM. Data from 5 independent experiments is presented in panels **B**, **C**, **E** and **F**

### MSCs transfer mitochondria to HNT CD4^+^ T cells

We sought to assess whether MSC-A were capable of transferring mitochondria to HNT CD4^+^ T cells when in co-culture. For this purpose, MSC-A were labeled with Mitotracker vital dye, which specifically stains mitochondria, and co-cultured with activated HNT CD4^+^ T cells. After 12 h, activated HNT CD4^+^ T cells were harvested and FACS analysis of Mitotracker fluorescence showed that they had acquired MSC mitochondria (Fig. 1E). As a control for Mitotracker leakage, activated HNT CD4^+^ T cells were cultured with supernatants from Mitotracker-labeled MSCs that had been processed as in the co-cultures (Fig. 1E). This control showed that our experimental conditions lead to some fluorescent Mitotracker leakage from MSCs, which remained nevertheless negligible compared to the mitochondria fluorescence signal acquired by T cells during coculture with MSCs (Fig. 1E). In addition, confocal microscopy confirmed the presence of Mitotracker-labeled MSC mitochondria in the cytoplasm of HNT CD4^+^ T cells (Fig. 1F). Our results demonstrate that the vast majority of HNT CD4^+^ T cells had acquired mitochondria from neighboring MSCs during co-culture.

### MSC mitochondria suppress HNT CD4^+^ T cell responses

In order to study the effects of MSC mitochondrial transfer, we have previously devised a method, termed “mitoception”, via which mitochondria isolated from MSCs are transferred into target cells [45, 49]. Different cell types, including tumor cells and human CD4^+^ Th17 cells, internalize mitocepted MSC mitochondria. Furthermore, mitoception mimicked some of the effects of MSC co-culture on these cells [45, 49]. Therefore, we utilized this method to evaluate the effects of MSC-A mitochondria on HNT CD4^+^ T cells. To validate the process, we first isolated mitochondria from 4 × 10^4^ Mitotracker-labeled MSC-A and utilized them to mitocept 10^6^ HNT CD4^+^ T cells to keep the same ratio used in co-culture experiments (1:25, MSC:T cells). CD4^+^ T cells were analyzed by FACS 12 h after mitoception and consistently showed uptake of Mitotracker-labeled MSC mitochondria (Fig. 2A). Confocal microscopy experiments clearly demonstrated that transferred mitochondria had been internalized by HNT CD4^+^ T cells (Fig. 2B). Further functional assays were performed in the same conditions but with non-labeled MSC-A mitochondria. Mock mitocepted controls were obtained by following the same mitoception protocol in the absence of mitochondria. After transfer of MSC mitochondria, CD4^+^ T cells were activated with anti-CD3 and anti-CD28 mAbs and cultured during 4 days. CD4^+^ T cells were enumerated and analyzed for their activation status. Similar to what was observed in co-cultures, acquisition of isolated MSC mitochondria promoted a significant decrease in CD4^+^ T cell expansion (Fig. 2C). There was a modest but significant decrease in the proliferation index accompanied by significant increases in the proportion of undivided cells and 7AAD^+^ cells (Fig. 2D). Additionally, a significant reduction in the percentage of IFNγ-producing cells was observed (Fig. 2E). On the other hand, we found no differences in CD40L expression and a minimal effect in CD25 expression (Fig. 2F). Our results demonstrate that MSC mitochondria inhibit the expansion and gain of effector function of HNT CD4^+^ T cells after activation.

### MSC mitochondria cooperate with soluble factors in the suppression of HNT CD4^+^ T cell responses

**Fig. 3:**
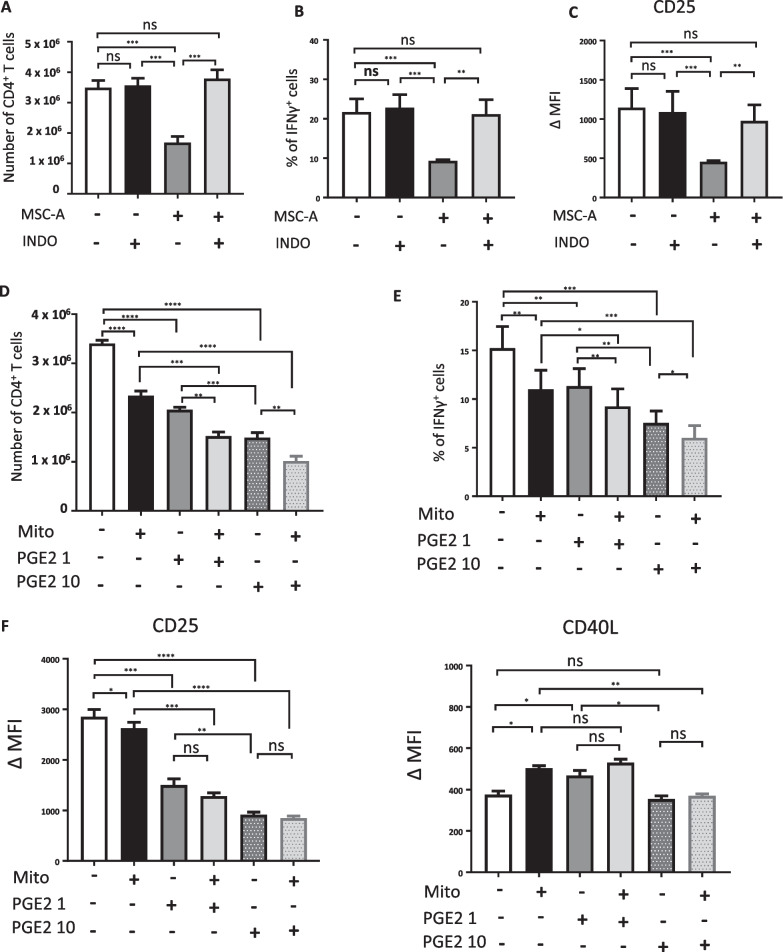
MSC mitochondria and PGE2 cooperate in the suppression of HNT CD4^+^ T cell responses. **A** Purified HNT CD4^+^ T cells were activated with anti-CD3 and anti-CD28 mAbs and cultured in the presence of MSC-A or absence. Indomethacin or vehicle was added to tissue culture media and cells cultured for 4 days. Absolute numbers of harvested T cells. **B** Day 4 activated HNT CD4^+^ T cells were restimulated with PMA and ionomycin in the presence of brefeldin A and production of intracellular IFNγ was assessed by FACS. Percentage of cytokine producing T cells is indicated. **C** CD25 expression on day 4 activated T cells was assessed by FACS. Mean fluoresce intensity (MFI) minus that of the isotype controls is indicated. **D** Isolated mitochondria from MSC-A were transferred by mitoception to naïve HNT CD4^+^ T cells. 12 h later, mitocepted or mock mitocepted HNT CD4^+^ T cells were activated with anti-CD3 and anti-CD28 mAbs and cultured during 4 days in the presence or absence of PGE2 (at a concentration of 1 or 10 ng/ml). Absolute numbers of harvested HNT CD4^+^ T cells. **E** Day 4 activated HNT CD4^+^ T cells were restimulated with PMA and ionomycin in the presence of brefeldin A and production of intracellular IFNγ was assessed by FACS. Percentage of cytokine producing T cells is indicated. **F** Expression of CD25 and CD40L in activated HNT CD4^+^ T cells. Mean fluorescence intensity (MFI) minus that of the isotype controls is indicated. Values are represented as mean ± SEM. Data from 3 independent experiments is presented in panels **A** to **F**

MSCs secrete a wide range of immunosuppressive cytokines and molecules. Among those, PGE2 has been shown to play a key role in the immunosuppressive effects of MSCs on CD4^+^ T cell responses. Synthesis of PGE2 depends on the activity of Cyclooxygenase (COX) enzymes and COX-specific inhibitors prevent PGE2 production [58]. We used the COX inhibitor indomethacin to prevent PGE2 production in our MSC-A/HNT CD4^+^ T cell co-cultures. Indomethacin abrogated MSC-mediated suppression of T cell proliferation and gain of effector function (Fig. 3A–C). This indicated that PGE2 is required for the suppression of HNT CD4^+^ T cell responses by MSCs. We next investigated whether MSC mitochondria and PGE2 cooperate in the suppression of HNT CD4^+^ T cell responses. MSC mitocepted and mock mitocepted HNT CD4^+^ T cells were activated in the presence or absence of PGE2. We used two PGE2 concentrations, namely 1 ng/ml, close to that measured in culture supernatants of murine MSCs [59], and 10 ng/ml. We found that PGE2 at 1 ng/ml significantly inhibited T cell expansion and IFNγ production to a similar extent as mitoception did (Fig. 3D, E). On the other hand, PGE2 at 1 ng/ml induced a higher decrease on CD25 expression than mitoception (Fig. 3F). These effects were even higher when PGE2 was used at 10 ng/ml (Fig. 3D–F). PGE2 did not inhibit CD40L expression at any concentration tested, similar results to that obtained with MSC-A mitochondrial transfer (Fig. 3F). Whereas we did not find any synergistic immunosuppressive effects between mitochondria and PGE2, we did find an additive effect on the suppression of proliferation and IFNγ production (Fig. 3D–F).

**Fig. 4:**
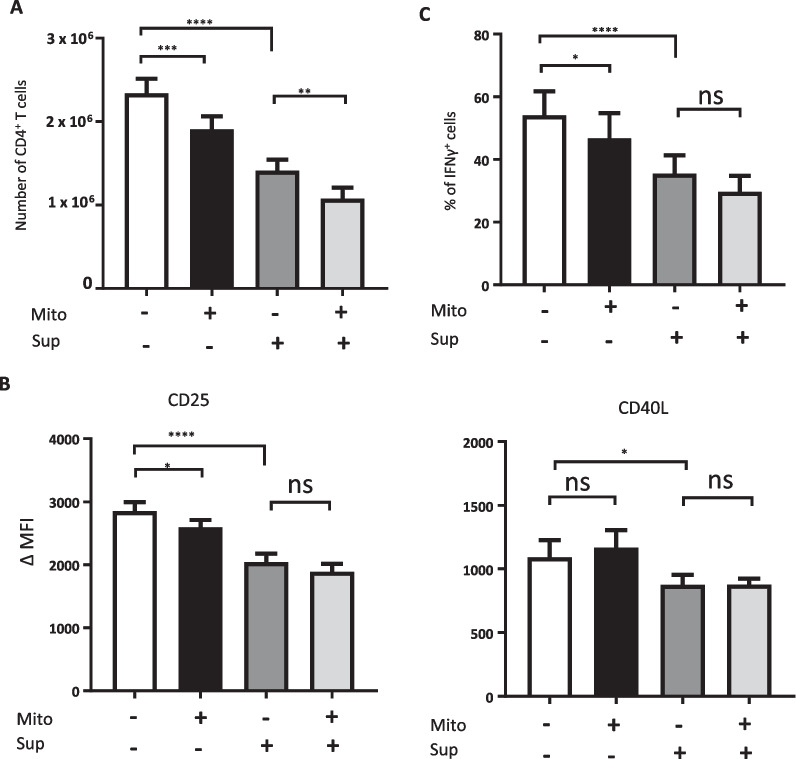
Additional soluble factors secreted by MSCs contribute to suppression of HNT CD4^+^ T cell responses. Isolated mitochondria from MSC-A were transferred by mitoception to naïve HNT CD4^+^ T cells. 12 h later, mitocepted or mock mitocepted HNT CD4^+^ T cells were activated with anti-CD3 and anti-CD28 mAbs and cultured during 4 days in the presence of MSC conditioned media (Sup) or fresh MSC media. Data from 4 independent experiments is presented. **A** Absolute numbers of harvested HNT CD4^+^ T cells. Values are represented as mean ± SEM. **B** Expression of CD25 and CD40L in activated HNT CD4^+^ T cells. Mean fluoresce intensity (MFI) minus that of the isotype controls is indicated. Values are represented as mean ± SEM. **C** Activated HNT CD4^+^ T cells were restimulated with PMA and ionomycin in the presence of brefeldin A and production of intracellular IFNγ was assessed by FACS. Percentage of cytokine-producing T cells is indicated. Values are represented as mean ± SEM

Neither MSC-A mitochondria nor PGE2 seemed to be involved in downregulation of CD40L. Thus, to assess whether different soluble factors secreted by MSC-A could account for the CD40L downregulation observed in co-cultures, we next tested the suppressive effect of MSC culture supernatants on HNT CD4^+^ T cell activation. We found that MSC-A conditioned media had similar effects that PGE2 on HNT CD4^+^ T cell proliferation, IFNγ production and CD25 expression (Fig. 4). Interestingly, we found that MSC-A supernatant was able to significantly inhibit CD40L upregulation (Fig. 4B). Again, we did not observe any synergistic effects of conditioned media with MSC-A mitochondrial transfer (Fig. 4). Our results indicate that additional factors other than PGE2 and exchanged mitochondria are required to account for the immunosuppressive effects of MSC-A on HNT CD4^+^ T cell differentiation. Altogether our results revealed that mitochondrial transfer, PGE2 and additional soluble factors cooperate and contribute to MSC-mediated immunosuppression.

### MSCs and MSC mitochondria prevent the upregulation of T-bet in activated HNT CD4^+^ T cells

**Fig. 5:**
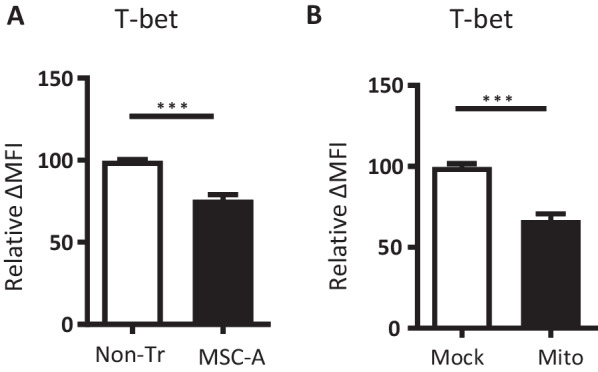
MSCs and MSC mitochondria prevent T-bet upregulation in activated HNT CD4^+^T cells. **A** Purified HNT CD4^+^ T cells were activated with anti-CD3 and anti-CD28 mAbs and cultured in the presence of MSC-A or left untreated without MSCs. After 24 h, CD4^+^ T cells were harvested and the expression of intracellular T-bet was analyzed by FACS. Mean fluorescence intensity (MFI) minus that of the isotype control relative to the non-treated control is indicated. Values are represented as mean ± SEM. Data from 4 independent experiments is presented. **B** Isolated mitochondria from MSC-A were transferred by mitoception to HNT CD4^+^ T cells. 12 h later, mitocepted or mock mitocepted HNT CD4^+^ T cells were activated with anti-CD3 and anti-CD28 mAbs and cultured during 24 h. Expression of intracellular T-bet was analyzed by FACS. Mean fluorescence intensity (MFI) minus that of the isotype controls relative to mock control is indicated. Values are represented as mean ± SEM. Data from 3 independent experiments is presented

IFNγ-producing CD4^+^ Th1 cell differentiation is dependent on the master transcription factor T-bet [60]. T-bet is upregulated upon antigen encounter and drives the expression of multiple genes involved in Th1 effector differentiation and in particular IFNγ [61]. Thus, we hypothesized that T-bet expression modulation could be a mechanism involved in suppression of HNT CD4^+^ T cell responses by MSC-A mitochondria. First, we compared T-bet expression in activated CD4^+^ T cells cultured either in the presence or absence of MSC-A by intracellular staining one day after activation. As shown by FACS analysis, T-bet expression was strongly upregulated in activated HNT CD4^+^ T cells (Fig. 5A and Additional file 1: Fig. 3A and B) and co-culture with MSC-A significantly inhibited this upregulation (Fig. 5A and Additional file 1: Fig. 3A and B). Finally, we assessed T-bet expression in activated CD4^+^ T cells that had been previously mitocepted with MSC-A mitochondria. Notably, we found a highly significant decrease of T-bet expression in these cells compared to control activated CD4^+^ T cells that did not receive mitochondria (Fig. 5B and Additional file 1: Fig. 3C). Our results demonstrate that both co-culture with MSC-A and transfer of isolated MSC-A mitochondria repress T-bet upregulation after HNT CD4^+^ T cell activation.

**Fig. 6:**
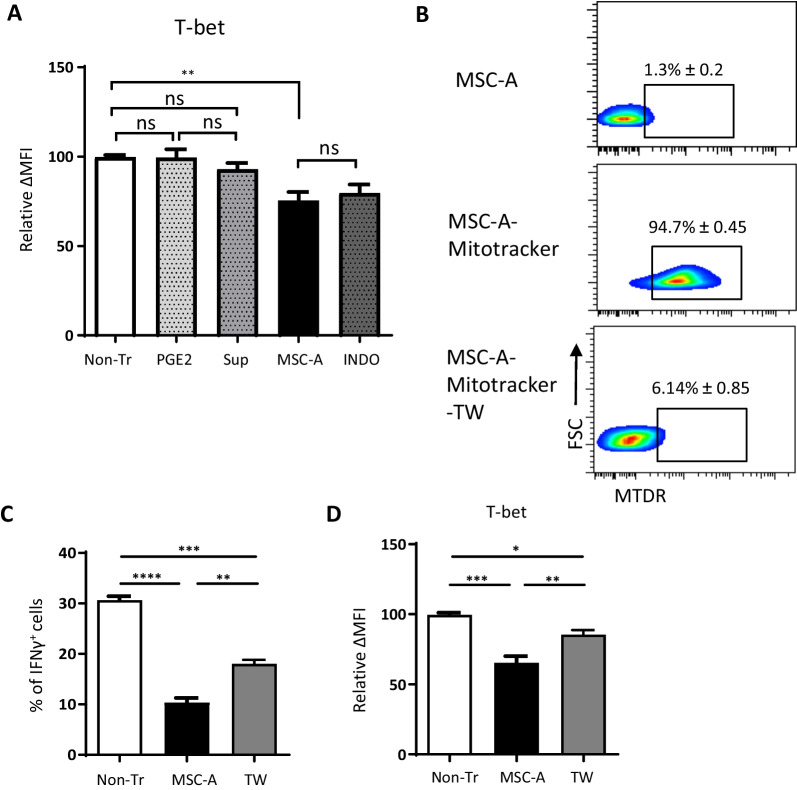
The role of MSC secreted soluble factors and cell–cell contact in T-bet expression. **A** Purified HNT CD4^+^ T cells were activated with anti-CD3 and anti-CD28 mAbs and cultured in the presence of PGE2 at 1 ng/ml (PGE2), MSC-A conditioned media (Sup), MSC-A, MSC-A plus indomethacin (INDO) or left untreated without MSCs (Non-Tr). After 24 h, CD4^+^ T cells were harvested and the expression of intracellular T-bet was analyzed by FACS. Mean fluorescence intensity (MFI) minus that of the isotype control relative to the non-treated control is indicated. Values are represented as mean ± SEM. Data from 3 independent experiments is presented. **B** Purified HNT CD4^+^ T cells were activated with anti-CD3 and anti-CD28 mAbs and cultured in the presence of MSC-A, Mitotracker Deep Red-labeled MSC-A or Mitotracker Deep Red-labeled MSC-A in a trans-well insert (TW). After 12 h, harvested T cells were analyzed by FACS to assess Mitotracker Deep Red (MTDR) fluorescence. Data from one representative experiment is presented. Values represent percentage of Mitotracker Deep Red^+^ cells ± SEM. **C** Purified HNT CD4^+^ T cells were activated with anti-CD3 and anti-CD28 mAbs and cultured either alone (Non-Tr), in the presence of MSC-A or MSC-A in a trans-well insert (TW). After 4 days, activated HNT CD4^+^ T cells were restimulated with PMA and ionomycin in the presence of brefeldin A and production of intracellular IFNγ was assessed by FACS. Percentage of cytokine-producing T cells is indicated. Values are represented as mean ± SEM. **D** Purified HNT CD4^+^ T cells were activated with anti-CD3 and anti-CD28 mAbs and cultured either alone (Non-Tr), in the presence of MSC-A or MSC-A in a trans-well insert (TW). After 24 h, CD4^+^ T cells were harvested and the expression of intracellular T-bet was analyzed by FACS. Mean fluorescence intensity (MFI) minus that of the isotype control relative to the non-treated control is indicated. Values are represented as mean ± SEM. Data from 3 independent experiments is presented in panels **A** to **D**

Next, we investigated whether soluble factors secreted by MSC-A have any role in T-bet downregulation. Activated HNT CD4^+^ T cells were cultured either in the presence of PGE2 or MSC-A culture supernatant. T-bet expression was unchanged after 24 h of culture (Fig. 6A). Furthermore, indomethacin was not able to revert MSC-A-mediated T-bet downregulation in activated HNT CD4^+^ T cells (Fig. 6A). These results indicate that soluble factors do not contribute to the modulation of T-bet expression by MSCs. Finally, we assessed T-bet expression under conditions where MSC-A mitochondrial transfer to T cells was hindered. The most common mechanism via which MSCs export their mitochondria is tunneling nanotubes and it requires contact with neighboring cells. Thus, co-culture of activated HNT CD4^+^ T cells with MSC-A in a trans-well setting almost completely abrogated mitochondrial transfer (Fig. 6B). Under these conditions, MSC-A mediated inhibition of IFNγ production by HNT CD4^+^ T cells was partially reverted (Fig. 6C). Notably, in trans-well co-cultures MSC-A-mediated T-bet downregulation was also reverted (Fig. 6D). These results suggest that in the absence of MSC mitochondrial transfer inhibition of T-bet expression and IFNγ production by MSC-A is prevented. Taken together our results show that MSC-A and MSC mitochondria inhibit the differentiation of CD4^+^ T cells into IFNγ producing Th1 cells and suggest that T-bet downregulation contributes to this effect.

## Discussion

Here we report that bone marrow derived allogeneic MSCs transfer mitochondria to CD4^+^ Th1-like cells and suppress their activation in response to TCR and co-stimulation signaling. IFNγ and TNFα-treated MSCs demonstrated a wide range of effects on activated HNT CD4^+^ T cells, including decreased proliferation, enhanced apoptosis, compromised upregulation of activation markers, decreased effector cytokine IFNγ secretion and a diminished diabetogenic potential in vivo. Notably, we found that MSC mitochondrial transfer contributed to suppression of Th1 responses. Analyzed as a separate event, transfer of MSC mitochondria to CD4^+^ T cells efficiently suppressed their expansion, prevented T-bet upregulation and IFNγ secretion. Importantly, these effects were reverted when mitochondrial transfer was hindered. These results, taken together with our previous observations on the suppression of human Th17 cells and the induction of Tregs by MSC mitochondria [45], provide evidence that MSC mitochondrial transfer represents a novel general mechanism of MSC immunosuppression.

It is well established that MSCs can suppress the expansion of T cells activated by antigen encounter [6, 12]. Here we show that MSC mitochondrial transfer contributes to this effect. Indeed, we found that MSCs and MSC mitochondria suppressed the expansion of HNT CD4^+^ T cells through two distinct mechanisms. First, they interfere with activated T cell proliferation, as evidenced by the increase in the number of undivided cells and cells that divided only a few times. Interestingly, a recent report showed that murine MSCs promote cyclin-dependent kinase 2 (CdK2) downregulation and cyclin-dependent kinase inhibitor 1B (CDKN1B) upregulation, resulting in cell cycle arrest and suppression of T cell proliferation [62]. Second, we found enhanced mortality in proliferating HNT CD4^+^ T cells. Although early studies did not find compromised T cell survival in the presence of MSCs [14], our data are in agreement with a study by Zhao et al. that showed an increased apoptotic rate in T cells co-cultured with human MSCs [33]. Both human and mouse MSCs are able to directly induce T cell apoptosis mediated by FasL expression [32, 33]. Interestingly, Li et al*.* reported that human MSCs promoted both cell-cycle arrest and apoptosis of T cells in an IDO-dependent manner [63]. Contrary to that, quiescent MSCs have been shown to promote survival of naïve T cells [9]. This provides evidence of the physiological homeostatic role of MSCs in the steady state, supporting components of the immune system in the bone marrow and peripheral tissues. However, under inflammatory conditions in vivo, or in the presence of TNFα and/or IFNγ in vitro, activated MSCs are able to suppress activated T cells as shown here.

We found that MSC mitochondria had an additive immunosuppressive effect with PGE2, at a concentration close to that regularly found in MSC supernatants, in the activation of HNT CD4^+^ T cells. In our studies, these additive effects were observed on T cell expansion and IFNγ production, where both mechanisms contributed to achieve inhibitory levels similar to those exerted by MSC co-culture. PGE2 is known to bind its receptors, EP2 and EP4, and interfere with TCR signaling in T cells, resulting in defective proliferation [64]. PGE2 has been found to suppress Th1 and Th17 cell proliferation and differentiation while promoting Th2 responses and inducing Tregs [16, 34, 65, 66]. Furthermore, PGE2 can directly inhibit IFNγ, IL-17 and other inflammation-related genes transcription [21]. Mitochondria had a minimal effect, if any, on the activation markers analyzed. On the other hand, PGE2 was able to efficiently inhibit CD25 expression upregulation, but not CD40L. Interestingly, CD40L expression was modulated by a different soluble factor, or alternatively a factor carried by extracellular vesicles, still to be identified. Our results demonstrate that the profound suppressive effects of MSCs on T cell activation are an extremely complex phenomenon to which multiple factors contribute. Although some of these factors have overlapping effects, each of them also appears to affect unique pathways and display a particular immunosuppressive signature that contributes to the overall MSC immunosuppressive effect.

MSCs are able to transfer mitochondria to a variety of cell types in different pathological contexts, as shown both in vitro and in vivo [39]. When there is tissue injury, therapeutic MSCs interact with damaged cells and transfer them their own mitochondria, exerting protective and reparatory effects [39, 40]. Similarly, transfer of MSCs mitochondria to tumor cells of different origins enhances their proliferation, invasiveness and resistance to chemotherapy [39, 41]. Although the fine mechanisms regulating the observed response in each particular case remain unclear, there are common features described in many of the models studied. That is, restoration of damaged mitochondrial metabolism, increased mitochondrial biogenesis and enhanced oxidative phosphorylation [39–41]. The immunomodulatory effects of MSCs on many different immune cell types have been extensively studied [12, 67]. However, the role of mitochondrial transfer in immunosuppression has just started to be explored. Mouse and human macrophages that received mitochondria from MSCs showed decreased inflammatory cytokines production mediated by a mitochondrial respiration enhancement [44]. For T cells, we have recently shown that transfer of MSC mitochondria to human Th17 cells compromised their effector function, promoted their conversion into FoxP3^+^ Tregs and also correlated with enhanced mitochondrial respiration [45]. Here, our results showed that mitochondrial transfer suppressed Th1 responses and downregulated T-bet expression. T-bet, the master regulator of Th1 differentiation, is firstly induced upon TCR signaling and then also regulated by IFNγ and IL-12 signaling [68–70]. T-bet directly regulates IFNγ expression and that of a number of chemokines, chemokine receptors, effector molecules and transactivators specific of Th1 cells. It also represses Th2, Th17 and TFH fates [69]. Thus, downregulation of T-bet might be responsible for the decrease in IFNγ production induced by both MSCs and MSC mitochondria. MSC mitochondrial transfer also inhibited T cell expansion in our studies. Naïve T cell activation and subsequent proliferation is thought to be dependent on a metabolic switch from a catabolic to an anabolic state with high glycolytic activity. This switch is supported by TCR and IL-2 signaling via the mTORC1 axis [71]. Notably, although the Th1, Th2, Th17 and TFH lineages have particular metabolic features, all share an anabolic metabolism with high glycolytic activity mediated by mTORC1 [60]. Interestingly, it has recently been shown that immunosuppressive MSCs inhibit T cell activation by disrupting mTOR signaling and in turn aerobic glycolysis [72]. Thus, in light of these results, it is interesting to speculate that MSC mitochondria suppress T cell proliferation by interfering with the mTORC1 pathway, favoring oxidative phosphorylation and preventing glycolysis.

The amount of MSC mitochondria internalized by HNT CD4^+^ T cells during mitoception, as visualized by the Mitotracker fluorescence intensity, appear to be much lower than during a 12 h co-culture period. This is likely due to the fact that physiological transfer is more efficient than artificial transfer and most importantly that in mitoception there is a single transfer event while during co-culture a continued transfer or multiple transfer events may occur. Thus, it is likely that the immunosuppressive effect seen after mitoception underrepresents the effect of mitochondria during the co-culture. Mitochondrial uptake by mouse CD4^+^ T cells here was similar to that previously observed in human Th17 cells leading to immunosuppression [45]. In that report, donor mitochondrial DNA (mtDNA) could be discriminated and quantified by qPCR taking advantage of single nucleotide polymorphisms. After a 4 co-culture period, the amount of donor MSC mtDNA represented 0.3% of the endogenous T cell mtDNA [45]. Interestingly, in a similar set of experiments, it was shown that human MSCs had transferred mitochondria to MDA-MB-231 cancer cells representing up to 4% of the endogenous mitochondria after a 24 h period [49].

Our results in vivo in a transgenic mouse model of autoimmune diabetes confirm and emphasize the therapeutic potential of MSCs for autoimmune diabetes. It has already been shown that i.v. infusion of MSCs in NOD mice, which develop autoimmune diabetes spontaneously, delays the onset of or reverts existing disease [73–75]. MSCs migrated preferentially to the draining lymph nodes of the pancreas and promoted a decrease in effector cytokines, a switch to a Th2 response and induction of Tregs [73, 75]. Thus, it is likely that, in our model, transferred MSCs are able to interact with diabetogenic CD4^+^ and CD8^+^ T cells in the pancreatic lymph nodes further suppressing their activation in response to beta cell-expressed HA antigen cross-presented by dendritic cells. Future studies will address whether these interactions occur and promote the exchange of mitochondria in vivo.

## Conclusion

Due to their strong immunosuppressive effects under inflammatory conditions, MSCs are currently studied as a cell-based therapy for autoimmune diseases as well as for disorders characterized by exacerbated inflammation. For this therapeutic strategy to be successful, it is essential to better understand the mechanisms underlying the immunomodulatory properties of MSCs in different pathogenic contexts and toward the variety of immune effectors involved. Here we have shown that allogeneic bone marrow-derived MSCs repress CD4^+^ T cell expansion and their differentiation into Th1 effectors in vitro, as well as their diabetogenic potential in vivo. Our data demonstrate that during intercellular interactions leading to immunosuppression, MSCs primed by pro-inflammatory cytokines transfer their mitochondria to activated Th1 cells. Notably, transfer of isolated MSC mitochondria to CD4^+^ T cells efficiently suppressed their expansion and secretion of IFNγ. Finally, we found that the mechanism via which MSC mitochondrial transfer repressed Th1 effector function is by downregulating expression of the master Th1 transcription factor T-bet. In conclusion, our data demonstrate that one important contributor of MSCs immunosuppressive properties is their mitochondrial export to target immune cells and provide evidence that it represents a general mechanism of MSC immunosuppression.

## Supplementary Information

**Additional file 1: Fig. S1** Flow cytometry data of HNT CD4^+^ T cells MSCs immunosuppression by MSCs in vitro.** Fig. S2** In vivo immunosuppressive potential of MSCs.** Fig. S3** Flow cytometry data of HNT CD4^+^ T cell T-bet expression.

## Data Availability

The datasets used and analyzed during the current study are available from the corresponding author on reasonable request.

## Abbreviations

COX: Cyclooxygenase
CTL: Cytotoxic T lymphocyte
DC: Dendritic cell
GVHD: Graft versus host disease
HA: Hemagglutinin
IDO: Indoleamine 2,3-doxigenase
iNOS: Inducible nitric oxide synthase
MSC: Mesenchymal stromal cell
MSC-A: Activated by TNFα and IFNγ MSC
mtDNA: Mitochondrial DNA
PGE2: Prostaglandin E 2
ROS: Reactive oxygen species
Tregs: Regulatory T cells
