# Supplementary material for: Transfer of mesenchymal stem cell mitochondria to CD4+ T cells contributes to repress Th1 differentiation by downregulating T-bet expression
Source: Stem Cell Res Ther. 2023 Jan 24;14:12. doi: 10.1186/s13287-022-03219-x (PMC9875419; doi:10.1186/s13287-022-03219-x)
Supplement: Supplementary file 1 — Additional file 1: Fig. S1 Flow cytometry data of HNT CD4+ T cells MSCs immunosuppression by MSCs in vitro. Fig. S2 In vivo immunosuppressive potential of MSCs. Fig. S3 Flow cytometry data of HNT CD4+ T cell T-bet expression. [file 13287_2022_3219_MOESM1_ESM.docx]

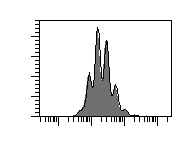

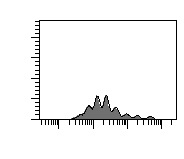

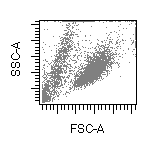

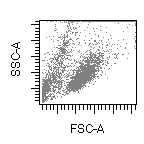

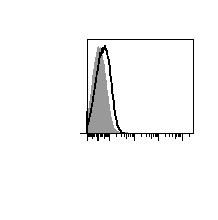

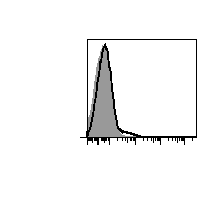

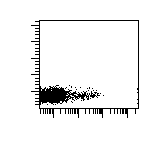

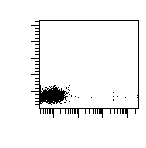

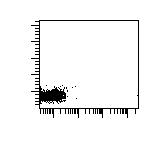

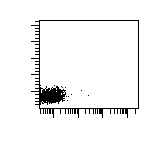

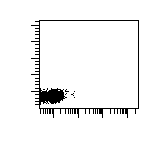

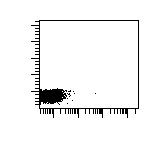

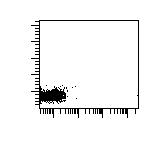

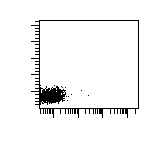

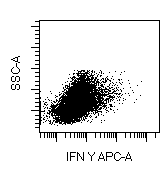

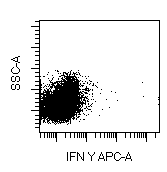

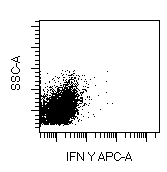

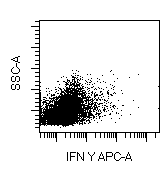

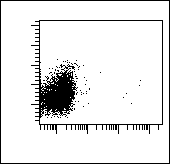

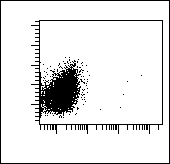

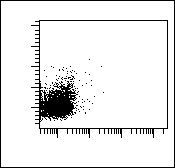

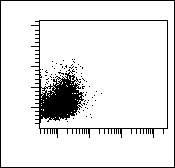

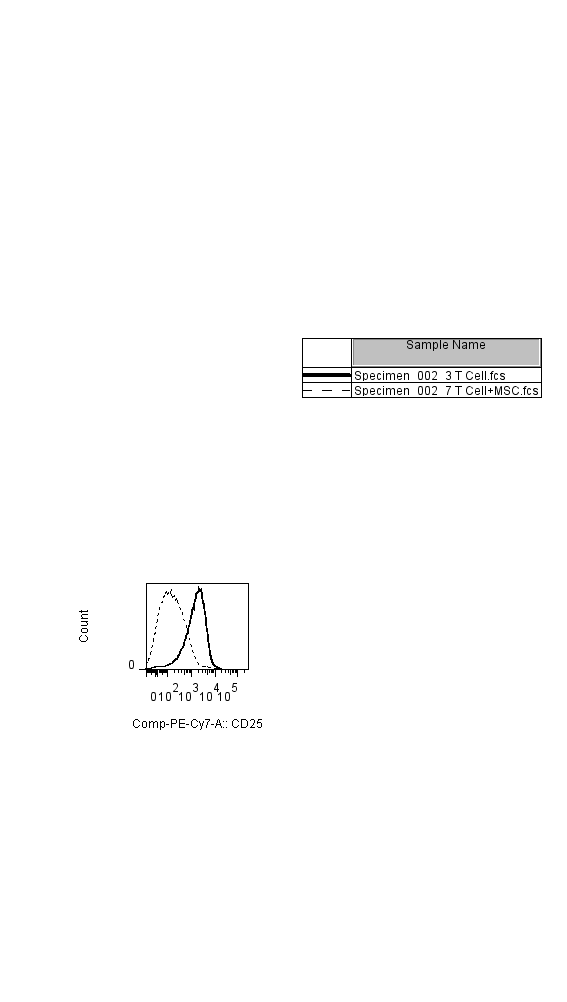

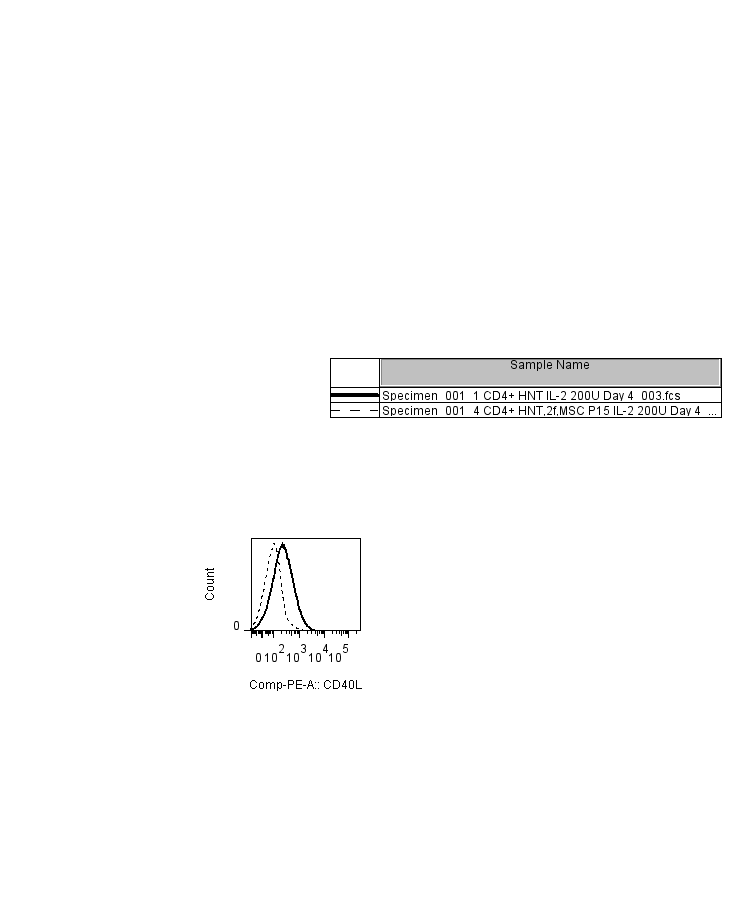


**Supp. Fig. 1**

**A**

**FSC**

**Non-Tr**

CFSE

**0.8%**

**B**

**CD25**

**CD40L**

**MFI**

**D**

**Naive**

**3.6%**

**0.2%**

**0.1%**

**0.1%**

**0.3%**

**0.5%**

**0.1%**

**0.1%**

**Isotype control**

**21%**

**0.8 %**

**Non-Tr**

**0.8 %**

**6.1 %**

**MSC-A**

**0.5 %**

**1.8 %**

**1.2 %**

**0.5 %**

**Isotype Control**

**Naive**

**Non-Tr**

**CD25**

**CD40L**

**Counts**

**C**

**E**

IFNγ

**IL-2**

**MSC-A**

**- - - -**

**Non-Stimulated**

**Stimulated**

**Non-Stimulated**

**Stimulated**

**SSC**

**SSC**

IFNγ

**IL-2**

**Non-Stimulated**

**Stimulated**

**Non-Stimulated**

**Stimulated**

**MFI**

**SSC**

**SSC**

**4.7 %**

**SSC**

**MSC-A**

**Supplemental figure 1**. **Allogeneic bone marrow derived MSCs suppress HNT CD4^+^ T cell responses. A**. CFSE-labeled naïve purified HNT CD4^+^ T cells were activated with anti-CD3 and anti-CD28 mAbs and cultured in the presence of C57Bl/6 bone marrow derived TNFα and IFNγ treated MSCs (MSC-A) or left untreated without MSCs (Non-Tr). After 4 days, CD4^+^ T cells were harvested and size and CFSE fluorescence analyzed by FACS. Values represent percentage of undivided cells. **B**. Expression of CD25 and CD40L in purified naïve HNT CD4^+^ T cells was analyzed by FACS. Data from one representative experiment out of two is presented. **C**. Day 4 activated HNT CD4^+^ T cells cultured in the presence or absence of MSC-A were harvested and expression of CD25 and CD40L was assessed by FACS. Data from one representative experiment out of 4, described in Fig. 1, is presented. **D**. Naïve HNT CD4^+^ T cells were stimulated with PMA and ionomycin in the presence of brefeldin A and production of intracellular IFNγ and IL-2 was assessed by FACS. **E**. Day 4 activated HNT CD4^+^ T cells were restimulated with PMA and ionomycin in the presence of brefeldin A and production of intracellular IFNγ and IL-2 was assessed by FACS. Percentage of cytokine producing T cells is indicated. Data from 4 independent experiments is presented. Values are represented as mean ± SEM.

**Supp. Fig. 2**

**A**

**Blood Glucose (mg/dl)**

**Days post transfer**

**B**

**CD4**

**CD4/MSC**

**CD4**

**CD4 + MSC In Vivo**

**CD4/MSC + MSC in Vivo**

**Supplemental figure 2. Allogeneic bone marrow derived MSCs delay the onset of T cell mediated autoimmune diabetes. A**. Sublethaly irradiated InsHA mice were adoptively transferred with 3x10^6^ naïve Clone 4 CD8^+^ T cells along with 3x10^6^ day 3 activated HNT CD4^+^ T (CD4) or 3x10^6^ day 3 activated HNT CD4^+^ T cells co-cultured with TNFα and IFNγ treated MSCs (CD4/MSC). Some CD4/MSC injected mice addtionally received 10^6^ MSCs on days 0 and 5 after transfer (CD4/MSC + MSC in vivo). Mice were monitored for the onset of autoimmune diabetes by measuring blood glucose levels. A bar is situated at the level of 300 mg/dl, concentration at which mice are considered diabetic. Data from two independent experiments is shown (n=6 mice per group). **B**. 3x10^6^ day 3 activated HNT CD4^+^ T cells and 3x10^6^ naïve Clone 4 CD8+ T cells were co-injected into sublethaly irradiated InsHA mice (CD4). Some mice additionally received 10^6^ MSC on days 0 and 5 after transfer (CD4 + MSC in vivo). Mice were monitored for the onset of autoimmune diabetes by measuring blood glucose levels. A bar is situated at the level of 300 mg/dl, concentration at which mice are considered diabetic. Data from two independent experiments is shown (n=4 mice per group).

**C**

**ns**

INDO

MSC-A

-

-

-

**% of IFNγ+ Cells**

+

+

+

-

**ns**


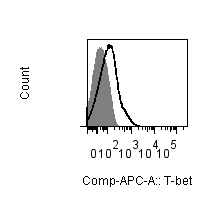


**Supp. Fig. 3**

**Tbet**

**Activated**

**Non-Tr**

**Activated**

**MSC-A**

**A**

**Tbet**

**Tbet**

**B**

**C**


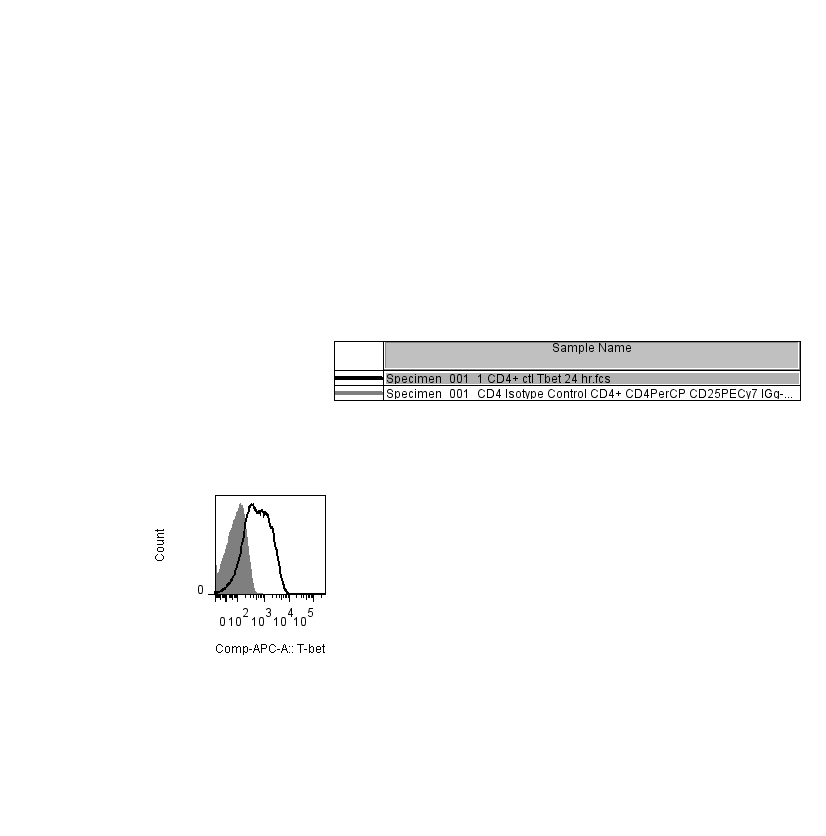

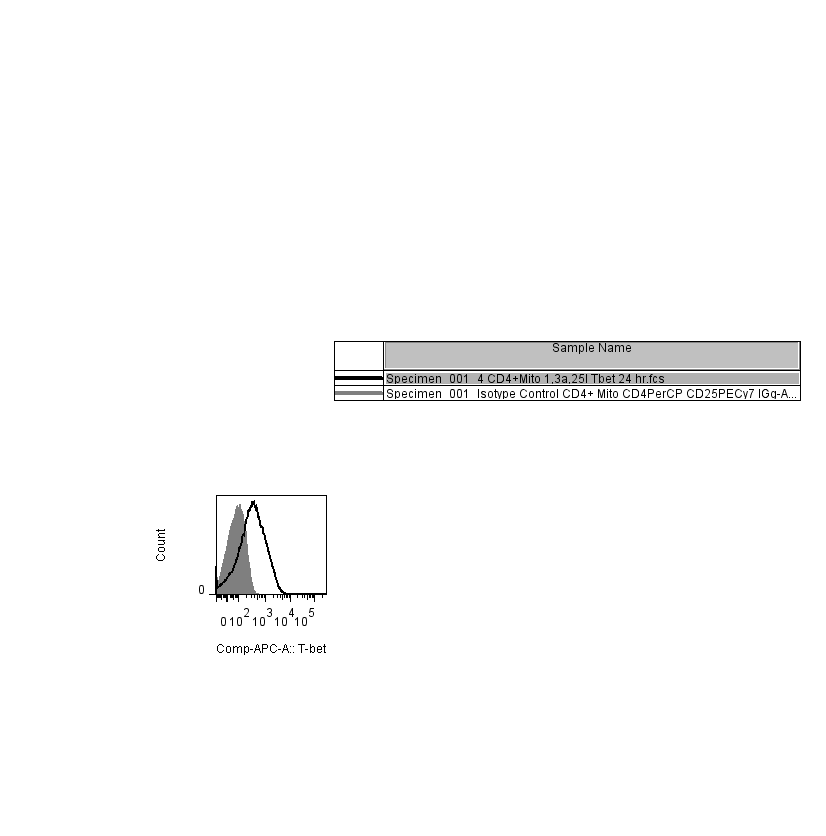

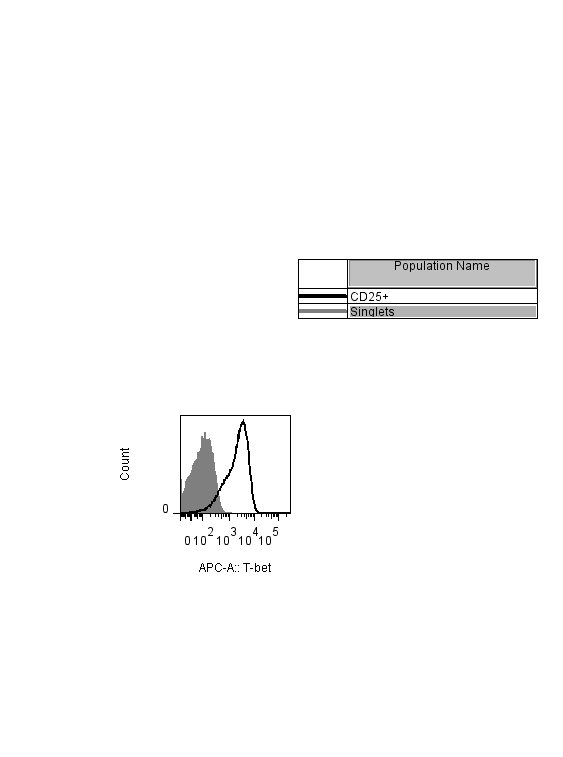

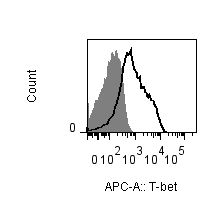


**Naive**

**Activated**

**Mito**

**Activated**

**Mock**

**Supplemental figure 4. MSCs and MSC mitochondria prevent upregulation of T-bet in activated HNT CD4^+^ T cells**. **A**. Expression of intracellular T-bet was analyzed in naïve purified HNT CD4^+^ T cells by FACS. **B**. Purified HNT CD4^+^ T cells were activated with anti-CD3 and anti-CD28 mAbs and cultured in the presence of TNFα and IFNγ treated MSCs (MSC-A) or left untreated without MSCs (Non-Tr). After 24h, CD4^+^ T cells were harvested and the expression of intracellular T-bet was analyzed by FACS**. C**. Naïve HNT CD4^+^ T cells were mitocepted with isolated mitochondria from TNFα and IFNγ treated MSCs or mock mitocepted without mitochondria. 12h later CD4^+^ T cells were activated with anti-CD3 and anti-CD28 mAbs and cultured during 24 h. Expression of intracellular T-bet was analyzed by FACS. Shaded histograms are Isotype-matched controls. Data from one out of 3-4 independent experiments is presented.
